# Exploring the Association of Autism Spectrum Disorders and Constipation through Analysis of the Gut Microbiome

**DOI:** 10.3390/ijerph18020667

**Published:** 2021-01-14

**Authors:** Shih-Chen Fu, Chung-Han Lee, Hsiuying Wang

**Affiliations:** Institute of Statistics, National Chiao Tung University, Hsinchu 30010, Taiwan; fushihchen@gmail.com (S.-C.F.); ww770829@yahoo.com.tw (C.-H.L.)

**Keywords:** autism spectrum disorders, constipation, microbiome, gut microbiome, bacteria, interaction

## Abstract

Over the past two decades, research into the role of the gut microbiome in regulating the central nervous system has rapidly increased. Several neurodevelopmental diseases have been linked to the unbalance of gut microbiota, including autism. Children on the autism spectrum often suffer from gastrointestinal symptoms, including constipation, which is four times more prevalent than it is in children without autism spectrum disorders (ASD). Although studies in animals have shown the crucial role of the microbiota in key aspects of neurodevelopment, there is currently no consensus on how the alteration of microbial composition affects the pathogenesis of ASD, let alone how it exerts an impact on the following comorbidities. In our study, we were able to control the effects of constipation on gut dysbiosis and distinguish neuropathological-related and gastrointestinal-related bacteria in ASD patients separately. By analyzing published data, eight additional bacteria significantly altered in autistic individuals were identified in our study. All of them had a decreased relative abundance in ASD patients, except Lactobacillaceae and Peptostreptococcaceae. Eighteen and eleven bacteria were significantly correlated with ASD symptoms and constipation, respectively. Among those, six bacteria were overlapped between the groups. We have found another six bacteria highly associated with constipation status in ASD patients only. By conducting Welch’s t-test, we were able to demonstrate the critical roles of microbes in ASD core and gastrointestinal symptoms and raised the hypotheses of their confounding and mediating effects on the relationship between the two symptoms.

## 1. Introduction

Autism spectrum disorders (ASD) are a group of complex disorders characterized by reduced social skills, restricted interests, and repetitive behaviors [1]. The prevalence of ASD is 1 in 36 children in the United States, among which boys are four times more likely to be diagnosed with ASD compared to girls [2,3]. Several comorbidities, including gastrointestinal (GI) symptoms, are frequently reported in autistic children [4,5]. Among those GI symptoms, constipation was observed to be the most common problem in ASD patients, with a prevalence of up to 50% or more [5,6,7,8]. The prevalence estimates of constipation in the general population were 24% and 39.6% only for chronic constipation and sub-chronic constipation, respectively [9].

Although the rate of ASD is rising, the causes are not well understood. Despite the fact that rare genetic variants have been found to contribute to the disease, they could only account for one third of all autistic cases [10]. Increasing evidence has shown that environmental factors play an important role in the etiology of ASD [11]. Recent studies have revealed differences between the gut microbiomes (the genomic profiles of gut microbiota) of ASD patients and healthy individuals, suggesting that ASD is associated with an unbalanced gut microbiota [12]. However, it is still not clear whether this unbalance, termed dysbiosis, is a factor causing ASD, or if the disease is causing the microbial alterations, let alone the exact microbial composition that is associated with the pathology of ASD and the following GI problems. Furthermore, although constipation is more prevalent in autistic children compared with normal individuals, the cause–effect relationship between ASD, constipation, and gut microbiota is not well established.

The aims of this study were to characterize microbes significantly correlating with ASD core symptoms and constipation separately, and to identify unique bacteria associated with constipation status in ASD patients. To achieve these purposes, we performed analyses on published data collected from autistic and non-autistic individuals, in which numbers of individuals suffering from constipation were clearly stated. We have found groups of bacteria that could reasonably explain the high prevalence of constipation among autistic individuals. Bacteria highly associated with constipation status in autistic, but not in neurotypical (NT), individuals were also identified. We further proposed hypotheses of confounding and mediating effects of the microbes in the interplay of ASD and constipation. One’s dietary habits and intestinal metabolic environmental changes could play a significant role in modulating the relation between ASD, gut microbiome, and constipation. Our results not only provide insights on the cause–effect relationship between ASD, constipation, and microbes, but also shed light on future microbiome-based therapies for alleviating or even preventing the symptom of constipation in autistic children.

## 2. Methods

We adapted our data from the study of Strati et al. [13], in which 40 autistic children (average age: 11.1 ± 6.8; gender: 31 males and 9 females) and 40 age and gender-matched neurotypical healthy subjects (average age: 9.2 ± 7.9; gender: 28 males and 12 females) were recruited. Details of individual characteristics, sample collection process, diagnostic criteria for ASD and constipation, and the measurement of microbiomes can be found in Appendix A and the previous reference [13]. Sample accessions IDs, metadata, and sequencing read information are available in Appendix B.

We downloaded results of mean relative abundance (%) ± standard deviation (SD) of bacterial taxa at genus levels in autistic and neurotypical subjects, both constipated and non-constipated, from Strati et al. [13]. We then conducted Welch’s t-test to investigate the difference of the relative abundance between groups. A *p*-value less than 0.05 is considered statistically significant. The Benjamini–Hochberg procedure was conducted to control the false discovery rate.

## 3. Results

**Figure 1:**
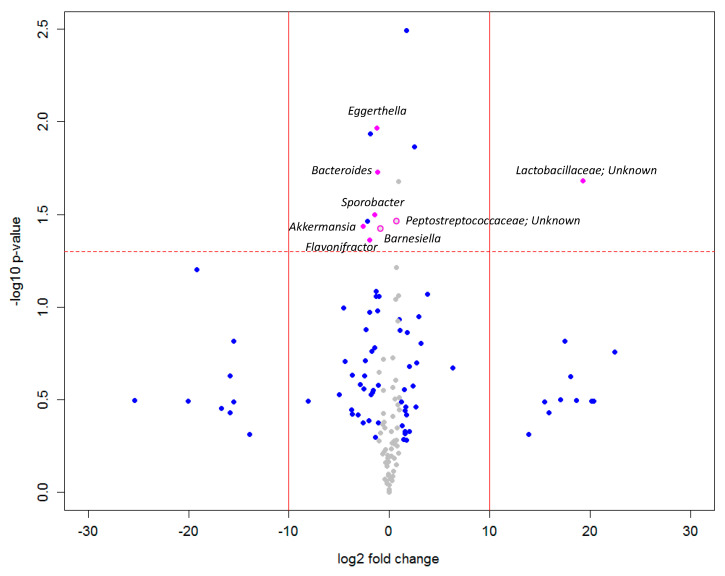
The overall difference of gut microbiota between autism spectrum disorders (ASD) and neurotypical (NT) individuals was assessed. A negligible number (0.000001) was added to the mean abundance of every bacterium to avoid the value of magnitude of fold change being infinitely large or small. This number was calculated based on the one hundredth of the minimal relative abundance in either group. Each point represents a bacterium with its magnitude fold change in relative abundance (log2 of ASD/NT) on the *x*-axis and the value of statistical significance (−log10 of *p* value) on the *y*-axis. The dashed red line shows where *p* = 0.05, with points above the line having *p* < 0.05 and points below the line having *p* > 0.05. Points with magnitude of fold change less than 1 are shown in grey. Points outside of the solid lines are bacteria with mean abundance of 0 in either ASD (left) or NT (right) group. Bacteria significantly altered in abundance in addition to the findings in Strati et al. were colored or circled in pink.

We first compared the difference of relative abundance of bacteria between ASD patients and healthy individuals. Out of 152 bacteria taxa detected in all subjects [13], 13 were found to be significantly different between the groups (Figure 1). Out of these 13 bacteria taxa, five were mentioned in the original study generated via LEfSe analysis [14]. The other eight bacteria taxa (*Eggerthella*, *Bacteroides*, Lactobacillaceae; Unknown, *Akkermansia*, Peptostreptococcaceae; Unknown, *Sporobacter*, *Flavonifractor*, *Barnesiella*), although not found in the original paper, have all been identified as autism-related bacteria in previous studies [12,15,16,17,18,19]. Notably, there was no Lactobacillaceae; Unknown detected in NT individuals, whereas the presence of this bacteria was found in ASD patients.

**Figure 2:**
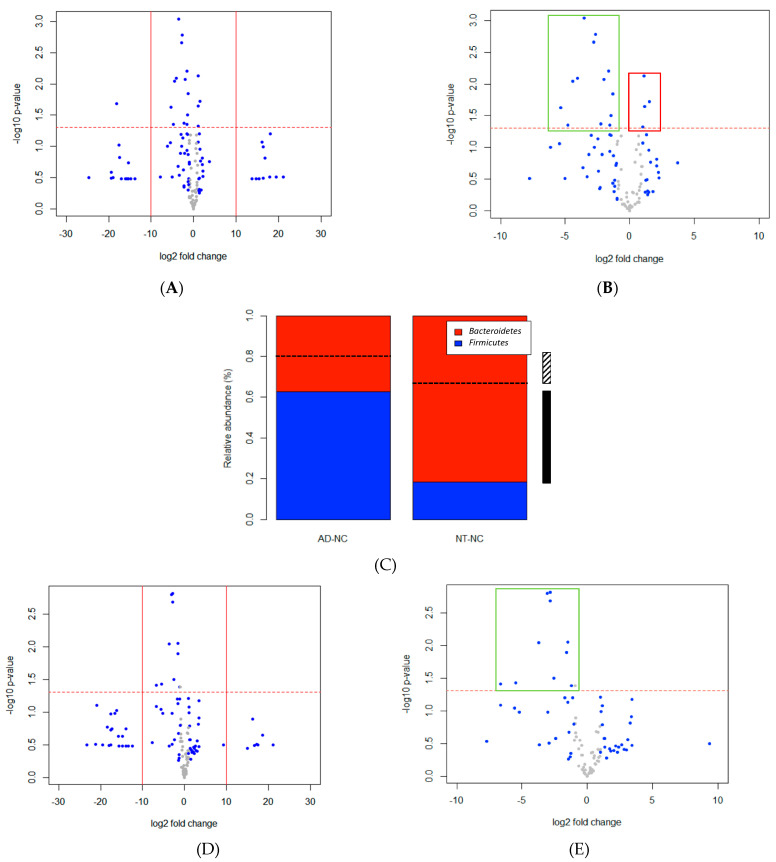
Volcano plots showing the differences of gut microbiota in (**A**) NT-NC versus AD-NC (Ga) and (**D**) NT-NC versus NT-C (Gc). A negligible number (Ga: 0.000002; NT-NC in Gc: 0.000002, NT-C in Gc: 0.000005) was added to the mean abundance of every bacterium to avoid the value of magnitude of fold change being infinitely large or small. The numbers were calculated based on one hundredth of the minimal relative abundance in each group. Each point represents a bacterium with its magnitude fold change (log2 of relative abundance) on the *x*-axis and the value of statistical significance (−log10 of *p* value) on the *y*-axis. The dashed red line shows where *p* = 0.05, with points above the line having *p* < 0.05 and points below the line having *p* > 0.05. Points having magnitude of fold change less than 1 are shown in grey. Points outside of the solid lines are bacteria with 0 abundance in one of the groups. Volcano plots of Ga (**B**) and Gc (**E**) with log2 fold change from −10 to 10 show degree of magnitude in relative abundance of bacteria substantially greater in decreasing (green box) than increasing (red box). (**C**) Mean relative abundances (%) of Firmicutes and Bacteroidetes in AD-NC and NT-NC subjects. Dashed lines show the approximate ratios of Firmicutes in Strati et al. when comparing ASD to NT. Differences of ratios of Firmicutes between groups are shown in stripy (AD vs. NT in Strati et al. [13]) and solid bars (AD-NC vs. NT-NC). AD: individuals with autism spectrum disorders; NT: neurotypical individuals; C: constipated; NC: non-constipated.

In order to understand which bacteria are highly associated with ASD core symptoms, we next compared the relative abundance of bacteria between the ASD and neurotypical individuals that were both free from constipation (non-constipated ASD individuals (AD-NC) vs. non-constipated neurotypical individuals (NT-NC)). Out of 152 bacteria genera detected in these two groups, 18 (hereinafter termed Ga) were significantly different in relative abundance between the two groups. By excluding the ones with unknown genera, 15 were left, in which 12 (80%) appeared to be lower in the ASD group (Figure 2A). The presence of *Sporobacter* in NT-NC was not seen in AD-NC. Interestingly, the degree of magnitude in decreasing of bacteria was substantially higher than increasing (Figure 2B). Phylum-level analysis showed a distinctive alteration of microbial composition in AD-NC characterized by a lower ratio of Bacteroidetes/Firmicutes (NT-NC = 4.46, AD-NC = 0.60). The proportion in relative abundances (%) of Firmicutes in these two phyla in AD-NC is 3.42 fold more than NT-NC (Figure 2C).

We then investigated the bacteria associated with constipation status in NT individuals only. When comparing the group of non-constipated and constipated NT subjects, the relative abundance of 11 bacteria taxa (hereinafter Gc) were found to be significantly different, suggesting that these imbalanced bacteria play a role in causing constipation (Figure 2D). All of the 11 bacterial taxa had a significant decrease in the NT constipated individuals (Figure 2E).

**Figure 3:**
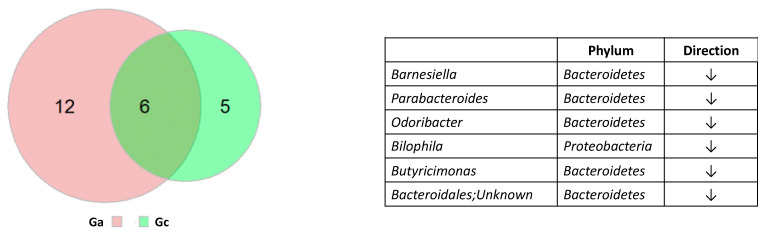
Six bacteria were overlapping between Ga and Gc. Size of the Venn diagram is shown in proportion to the number of bacteria in each group. Bacterial names and direction of changing relative abundance are shown on the right.

We next searched for bacteria that were highly associated with both ASD core symptoms and constipation. Six taxa (hereinafter Ga_c) were overlapping between Ga and Gc (Bacteroidales; Unknown, *Barnesiella*, *Parabacteroides*, *Odoribacter*, *Bilophila*, *Butyricimonas*). All of these bacteria belong to the phylum Bacteroidetes except *Bilophila*. All six bacterial taxa significantly decreased in relative abundance in both Ga and Gc (Figure 3).

**Figure 4:**
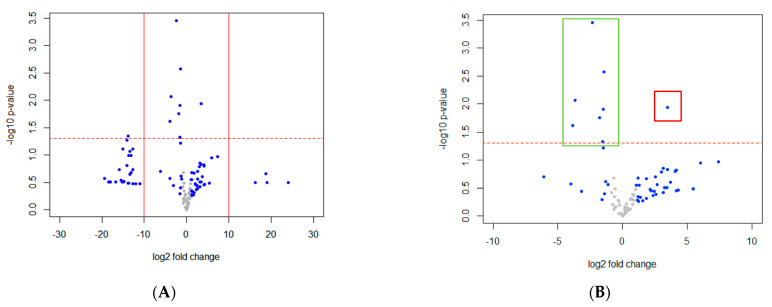
(**A**) Volcano plot showing the differences of gut microbiota in AD-NC versus AD-C (Gc_a). A small number (AD-NC: 0.000002, AD-C: 0.000012) was added to the mean abundance of every bacterium to avoid the value of magnitude of fold change being infinitely large or small. Each point represents a bacterium with its magnitude fold change (log2 of relative abundance) on the *x*-axis and the value of statistical significance (−log10 of *p* value) on the *y*-axis. The dashed red line shows where *p* = 0.05, with points above the line having *p* < 0.05 and points below the line having *p* > 0.05. Points having magnitude of fold change less than 1 are shown in grey. Points outside of the solid lines are bacteria with 0 abundance in one of the groups. (**B**) Volcano plot of log2 fold change from −10 to 10 shows degree of magnitude in relative abundance of bacteria substantially greater in decreasing (green box) than increasing (red box).

**Figure 5:**
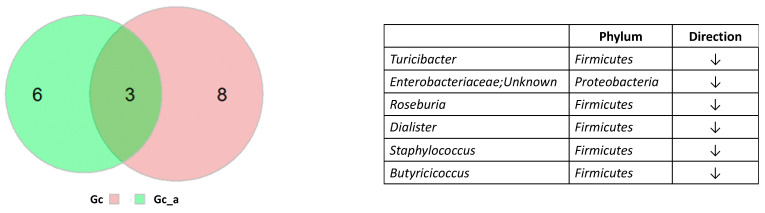
Six bacteria were unique in Gc_a comparing to Gc. Size of the Venn diagram is shown in proportion to the number of bacteria in each group. The 6 unique bacterial names and direction of changing relative abundance are shown on the right.

By comparing gut microbiota compositions in non-constipated and constipated ASD children (AD-NC vs. AD-C), we found nine bacteria taxa (Gc_a) showing significant difference in relative abundance (Figure 4A). The abundance was substantially lower in all bacteria except Enterobacteriaceae; Unknown in AD-C (Figure 4B). Seven out of the nine bacteria taxa had genus information and all belong to the Firmicutes phylum. Even after *p*-value correction using the Benjamini–Hochberg method, one of the bacteria (*Faecalibacterium*) was still significant. To further understand the bacteria correlating with the constipation status in ASD only, we removed Gc from Gc_a. Six imbalanced bacteria taxa were uniquely associated with constipation status in ASD patients (Gc_a_uniq), among which five had genus information (*Turicibacter*, *Roseburia*, *Dialister*, *Staphylococcus*, *Butyricicoccus*). These five bacteria genera belong to the Firmicutes phylum and all anticorrelate with constipation status (Figure 5). On the other hand, out of the eight imbalanced bacteria taxa that were uniquely associated with constipated NT individuals, five (62.5%) belong to the Bacteroidetes phylum, and only two (25%) were in the Firmicutes phylum. All of these bacteria anticorrelate with constipation status. The decreasing of *Faecalibacterium*, *Gemmiger*, and an unknown bacterium were associated with constipation status in both ASD and NT individuals (Figure 5).

## 4. Discussion

ASD is a complex neurological and developmental disorder often followed by various comorbidities, including GI symptoms. Studies have shown that autistic individuals harbor an altered bacterial gut microbiota [20,21,22,23]; however, there has been little consensus on specific bacterial species that were similarly altered across autistic individuals. Most studies were done without ruling out the effect existing comorbidities potentially had on the composition of the gut microbiome, which left the etiology of ASD elusive. To the best of our knowledge, we are the first to investigate the role gut microbiota play on ASD and its comorbidities independently. The data released by Strati et al. [13] enabled us to assess microbial populations associated with psychiatric impairment and GI symptoms separately.

**Figure 6:**
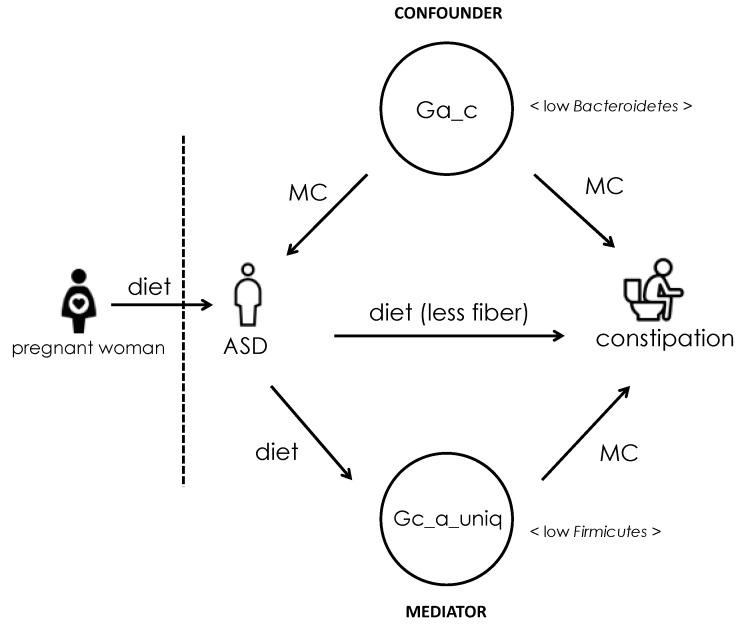
The hypothetical causal relations between ASD, gut microbes, and constipation. MC: metabolic change.

Among the eight additional bacterial taxa that were found correlating with the 40 ASD patients tested, family Lactobacillaceae was present in the patients but not found in NT individuals. This family has been observed as one of the key families in differentiating ASD from healthy children [24]. By analyzing the microbes in ASD and NT subjects with or without constipation status, bacteria specifically contributing to ASD symptoms, constipation, and both were identified. Several studies have shown that gut dysbiosis could lead to ASD-like and GI symptoms through independent mechanisms. The early-life gut microbiome could influence later neurodevelopment to a great extent in animal models. These bacteria secrete neurotransmitters which interferes with hosts’ central neural pathways [25]. Similarly, evidence from a human study showed a strong linkage between infant gut microbes and childhood neurodevelopmental outcomes [26]. Other evidence suggests that alterations of bacteria may also lead to GI symptoms by influencing intestinal motility and secretory functions through changing the metabolic environment in the gut [27,28]. Taking the above evidence together, we could reasonably hypothesize that Ga_c are risk factors for both ASD and GI symptoms (Figure 6). Due to the fact that ASD patients are at greater risk of constipation, the possibility that the presence of Ga_c positively confounds the association between ASD and constipation cannot be underestimated. The potential confounding effect of Ga_c may account for all or part of the association. Levels of the effect could be tested in animal models by adjusting their intestinal microbial composition.

We found that Bacteroidales; Unknown ranked third (*p* = 0.0022) among Ga. The fold change of its relative abundance was 0.14 in AD-NC compared to NT-NC. Bacteroidales; Unknown was also the second most significantly reduced bacteria in Gc (*p* = 0.0016). The fold change of its relative abundance in NT-C versus NT-NC was 0.12. That the lack of Bacteroidales could possibly lead to ASD and constipation can be supported by a study done by Hsiao et al. [29]. They demonstrated that introducing human commensal *Bacteroides fragilis* to a mouse model of ASD could reverse many of the behavioral and gastrointestinal changes. *Sporobacter* was another bacterial taxon that was significantly altered in Ga. In fact, it was completely absent in AD-NC. Multiple studies have shown *Sporobacter* significantly decreased in ASD children [12,30], indicating that the decreasing of *Sporobacter* could be one of the biomarkers for ASD core symptoms. Together with the findings provided by Strati et al., these results lead us a step closer to understanding the crosstalk between the central nervous system and the gut microbiota.

Several literatures have shown a decrease in the Bacteroidetes/Firmicutes ratio in the fecal samples of autistic children [13,31,32]. In our study, we have found that the alteration was even greater between AD-NC and NT-NC, indicating that bacteria within the Firmicutes phylum contributed substantially to the core symptoms of ASD. Firmicutes is known for its function of degrading polysaccharides and converting them into carbohydrates and other energy products [33]. Although carbohydrates serve as one of the main fuels for our brains, excess carbohydrates could cause numerous neurological conditions such as Alzheimer’s disease, Parkinson’s disease, depression, and others [34,35,36,37]. It is likely that an abnormal amount of Firmicutes could lead to a surplus amount of carbohydrates in the bloodstream, causing neurological and behavioral problems associated with ASD. This also explains the high rate of obesity among teens with autism [38] as a higher Firmicutes/Bacteroidetes ratio has been linked to excess body fat in previous studies [39,40,41].

The effects of microbial composition and ASD can be mutual. Not only can microbial dysbiosis contribute to ASD and constipation, but intestinal microbes could also be influenced through one’s dietary habits and further cause constipation. In other words, the causal effects of ASD and constipation are mediated by the altered microbes (Figure 6). Although the connection is poorly understood, diet does affect the composition of gut bacteria [42]. It is well known that most ASD patients are very sensitive to not just the flavor, but also the color, smell and texture of foods, resulting in a strong preference for a narrow selection of foods. Many prefer having carbohydrates and processed foods and dislike high-fiber food such as fruits and vegetables. This behavior may lead to lower beneficial Firmicutes and higher levels of mucosa-associated Proteobacteria (Figure 5) [43]. Previous studies have shown that mice fed with a high-fat diet showed a marked decrease of *Turicibacter* in abundance. In our study, the decreasing of *Turicibacter* was the most significant (*p* = 0.0086) among all bacteria associated with constipation status in ASD. *Dialister* was another bacterium with a significant decrease in constipated ASD patients compared to non-constipated ones. This difference was not found between healthy constipated and non-constipated individuals. The dislike of high-fiber food in ASD may result in the reduction of *Dialister*. A human study has demonstrated that a daily dose of 60 g of whole-grain barley (WGB) would enrich the genera of *Dialister* [44]. *Roseburia* and *Butyricoccus* were another two beneficial bacteria which were found significantly reduced in constipated ASD patients in our study. This again might be due to selective eating habits. One study found that a high-animal-protein diet, which is high in fat, could result in reduced *Roseburia* in the tested subjects [45]. It is worth noting that the dietary component of pregnant women also highly correlates with the health status of the baby. It has been suggested that the lack of Bacteroidetes may contribute to malnourishment via a reduction in ability to ferment glycans and generate short-chain fatty acids (SCFAs) [46]. Maternal malnutrition jeopardizes the neurodevelopment of the fetus, which could cause prematurity or growth restriction of the baby [47]. It has been shown that preterm babies have a significantly greater risk of ASD [48].

The published data adapted in our study was collected from an Italian research group in which stool samples were produced by individuals on Mediterranean-based diets. Information regarding a more detailed diet preference in ASD and healthy subjects was not available in the original article. Whether the diet one consumes could alter the ASD-specific signature which ultimately leads to constipation has yet to be analyzed. It is worth noting that subjects with different ethnicities could yield different results due to their unique genetics and dietary habits. One limitation of using summary data lies within the missing resolution on the individual level, making it difficult to conduct pathway analysis. It would be interesting to include this type of analysis in our future studies, which could possibly reveal the underlying mechanisms of how microbial functionalities’ change is associated with ASD and constipation. Another limitation resulting from using summary data is insufficient statistical power. Analysis of data on the individual level would be our next step to increase the power of our study. Lastly, the data analyzed in this study was generated from bacterial 16S rRNA, in which the resolution was at best at the genus level. Bacterial species under the same genus produce metabolites with various functions and might have different contributions to ASD and its comorbidities. In order to examine results at a higher resolution, analyzing metagenomic data would be a good approach.

## 5. Conclusions

To the best of our knowledge, we are the first to raise the hypotheses of confounding and mediating effects of gut microbiota to ASD and constipation. In summary, we were able to identify ASD-correlated bacteria which failed to be detected in the original study. Our results suggest multi-directional and complex interactions between ASD, microbiome, and constipation. Children with a low abundance of Bacteroidetes may have a higher chance of developing ASD and constipation simultaneously; however, further case-parent trio studies or comparisons between AD children and their NT siblings need to be done in order to confirm the causality. In the case of children who are already on the spectrum, low abundance of Firmicutes may be one of the major causes of constipation. In addition, the relative abundances of six unique diet-related bacteria were found to be significantly lower in constipated ASD subjects. Longitudinal studies are necessary to further confirm the relevance of these microbes in ASD and constipation through diet.

## Data Availability

No new data were created or analyzed in this study. Data sharing is not applicable to this article.

## Appendix A. Methods

### Appendix A.1. Population and Study Design

We adapted our data from the study of Strati et al., in which 40 autistic children (average age: 11.1 ± 6.8; gender: 31 males and 9 females) and 40 age and gender matched neurotypical healthy subjects (average age: 9.2 ± 7.9; gender: 28 males and 12 females) were recruited. Autistic subjects and their fecal samples were admitted and collected at the Child Neuropsychiatry Unit of the University Hospital of Siena. Subjects recruited in the study had not taken antibiotics, probiotics, or prebiotics in the past 3 months before sample collection and all of them were under consistent diet. Details can be found in the previous reference.

### Appendix A.2. Diagnostic Criteria for Autism Spectrum Disorder (ASD) and Constipation

The Diagnostic and Statistical Manual of Mental Disorders in 5th Edition (DSM-V), Autism Diagnostic Observation Schedule, and Autism Behaviour Checklist were used to diagnose and evaluate ASDs. Scores of Childhood Autism Rating Scale (CARS), which is a behavior rating scale developed to diagnose autism, were calculated by a highly trained child neuropsychiatrist. 36 (90%) autistic children were classified as severe ASDs and the other 4 (10%) were classified as moderately severe based on CARS scores. There were no comorbidities other than constipation found in the autistic cohort with an exception of celiac disease in 2 patients (5%). Rome III criteria was used to define constipation. The criteria must be fulfilled for the past 3 months, with onset of symptoms at least 6 months before diagnosis.

### Appendix A.3. Measurement of Microbiome

In order to understand the gut microbial composition in an individual, one’s fecal samples were collected and sent for total DNA extraction. Bacterial 16S rRNA genes were subsequently amplified by PCR using a specific primer set, which contains barcodes (unique sequences) that could ligate to each individual’s samples’ genetic material before samples were mixed together. Once the final amplicon library is constructed, high throughput DNA sequencing (454 pyrosequencing on the GS FLX+ system using the XL+ chemistry) was carried out by following the manufacturer’s instructions. The sequencing data obtained were demultiplexed (processing steps in which the barcode information shows which sequences came from which samples after being sequenced together) and submitted to the European Nucleotide Archive (ENA) with accession numbers PRJEB15418.

MICCA pipeline (v. 0.1) was used to preprocess the reads. The RDP classifier version 2.7 was used to assign reads to the corresponding taxonomy model. Template-guided multiple sequence alignment (MSA) was performed using PyNAST (v. 0.1) against the multiple alignment of the Greengenes database (release 13_05) filtered at 97% similarity.

For more details on total DNA extraction from stool samples, sequencing procedures on the bacterial 16S rRNA genes, and how the reads were processed were explained in the section of “Pyrosequencing and data analysis” under Methods of Strati et al., 2017.

### Appendix A.4. Statistical Analysis

We downloaded results of mean relative abundance (%) ± standard deviation (SD) of bacterial taxa at genus levels in autistic and neurotypical subjects both constipated and non-constipated from Strati et al., 2017. We then conducted Welch’s t-test to investigate the difference of the relative abundance between groups. A p-value less than 0.05 is considered as statistically significant.

### Appendix A.5. Ethics Approval and Consent to Participate

The data we have used in this analysis is publicly available, which can be downloaded from Strati et al., 2017 [13]. The original human study was approved by the Institutional Review Board of the Siena University Hospital (AOUS, Siena, Italy), and informed consent was obtained from either the parents or the legal tutors of the participants, in compliance with national legislation and the Code of Ethical Principles for Medical Research Involving Human Subjects of the World Medical Association (Declaration of Helsinki) [13].

## Appendix B. Read Number from the Minimum to the Maximum

	**ctrl**	**AD**
	1783	6
	2628	14
	3348	49
	3845	78
	4770	1578
	5165	1600
	5444	2176
	5628	3513
	7553	3577
	8525	3730
	8959	4217
	10,749	4266
	10,956	4520
	11,926	4745
	12,762	4856
	13,607	4880
	13,717	5366
	15,347	7307
	16,331	7596
	17,866	8214
	18,395	8934
	18,858	9299
	18,878	11,520
	18,935	11,642
	19,764	12,861
	19,931	13,814
	21,293	15,406
	21,410	15,529
	21,532	18,527
	21,548	19,308
	21,695	19,563
	23,142	21,200
	27,287	21,213
	29,311	22,653
	34,413	22,702
	37,965	27,421
	41,923	30,120
	42,749	33,036
	42,880	48,288
	46,018	72,830
**Average (AD w/o the first 4 samples)**	18,220.9	14,666.8611

## References

[1] Association A.P. (2013). Diagnostic and Statistical Manual of Mental Disorders (DSM-5®).

[2] Zablotsky B., Black L.I., Blumberg S.J. (2017). Estimated Prevalence of Children with Diagnosed Developmental Disabilities in the United States, 2014–2016. NCHS Data Brief.

[3] Christensen D.L., Braun K.V.N., Baio J., Bilder D., Charles J., Constantino J.N., Daniels J., Durkin M.S., Fitzgerald R.T., Kurzius-Spencer M. (2018). Prevalence and characteristics of autism spectrum disorder among children aged 8 years—autism and developmental disabilities monitoring network, 11 sites, United States. MMWR Surveill. Summ..

[4] Gorrindo P., Williams K.C., Lee E.B., Walker L.S., McGrew S.G., Levitt P. (2012). Gastrointestinal Dysfunction in Autism: Parental Report, Clinical Evaluation, and Associated Factors. Autism Res..

[5] Wang L.W., Tancredi D.J., Thomas D.W. (2011). The Prevalence of Gastrointestinal Problems in Children Across the United States with Autism Spectrum Disorders from Families with Multiple Affected Members. J. Dev. Behav. Pediatr..

[6] Buie T., Fuchs G.J., Furuta G.T., Kooros K., Levy J., Lewis J.D., Wershil B.K., Winter H. (2010). Recommendations for Evaluation and Treatment of Common Gastrointestinal Problems in Children with ASDs. Pediatrics.

[7] Coury D.L., Ashwood P., Fasano A., Fuchs G., Geraghty M., Kaul A., Mawe G., Patterson P., Jones N.E. (2012). Gastrointestinal Conditions in Children with Autism Spectrum Disorder: Developing a Research Agenda. Pediatrics.

[8] Holingue C., Newill C., Lee L.-C., Pasricha P.J., Fallin M.D. (2018). Gastrointestinal symptoms in autism spectrum disorder: A review of the literature on ascertainment and prevalence. Autism Res..

[9] Werth B.L., Williams K.A., Fisher M.J., Pont L.G. (2019). Defining constipation to estimate its prevalence in the community: Results from a national survey. BMC Gastroenterol..

[10] Yu L., Wu Y., Wu B.-L. (2015). Genetic architecture, epigenetic influence and environment exposure in the pathogenesis of Autism. Sci. China Life Sci..

[11] Karahmadi M., Karimi P., Kamali E., Mousavi S.M. (2017). Environmental factors influencing the risk of autism. J. Res. Med. Sci..

[12] Srikantha P., Mohajeri M.H. (2019). The Possible Role of the Microbiota-Gut-Brain-Axis in Autism Spectrum Disorder. Int. J. Mol. Sci..

[13] Strati F., Cavalieri D., Albanese D., De Felice C., Donati C., Hayek J., Jousson O., Leoncini S., Renzi D., Calabrò A. (2017). New evidences on the altered gut microbiota in autism spectrum disorders. Microbiome.

[14] Segata N., Izard J., Waldron L., Gevers D., Miropolsky L., Garrett W.S., Huttenhower C. (2011). Metagenomic biomarker discovery and explanation. Genome Biol..

[15] Xu M., Xu X., Li J., Li F. (2019). Association between Gut Microbiota and Autism Spectrum Disorder: A Systematic Review and Meta-Analysis. Front. Psychiatry.

[16] Ma B., Liang J., Dai M., Wang J., Luo J., Zhang Z., Jing J. (2019). Altered Gut Microbiota in Chinese Children with Autism Spectrum Disorders. Front. Cell. Infect. Microbiol..

[17] Wang M., Wan J., Rong H., He F., Wang H., Zhou J., Cai C., Wang Y., Xu R., Yin Z. (2019). Alterations in Gut Glutamate Metabolism Associated with Changes in Gut Microbiota Composition in Children with Autism Spectrum Disorder. mSystems.

[18] Liu S., Li E., Sun Z., Fu D., Duan G., Jiang M., Yu Y., Mei L., Yang P., Tang Y. (2019). Altered gut microbiota and short chain fatty acids in Chinese children with autism spectrum disorder. Sci. Rep..

[19] Coretti L., Paparo L., Riccio M.P., Amato F., Cuomo M., Natale A., Borrelli L., Corrado G., De Caro C., Comegna M. (2018). Gut Microbiota Features in Young Children with Autism Spectrum Disorders. Front. Microbiol..

[20] Fattorusso A., Di Genova L., Dell’Isola G.B., Mencaroni E., Esposito S. (2019). Autism Spectrum Disorders and the Gut Microbiota. Nutrients.

[21] I Kushak R., Buie T., Murray K.F., Newburg D.S., Chen C., Nestoridi E., Winter H.S. (2016). Evaluation of Intestinal Function in Children with Autism and Gastrointestinal Symptoms. J. Pediatr. Gastroenterol. Nutr..

[22] De Angelis M., Francavilla R., Piccolo M., De Giacomo A., Gobbetti M. (2015). Autism spectrum disorders and intestinal microbiota. Gut Microbes.

[23] Borre Y.E., O’Keeffe G.W., Clarke G., Stanton C., Dinan T.G., Cryan J.F. (2014). Microbiota and neurodevelopmental windows: Implications for brain disorders. Trends Mol. Med..

[24] Pulikkan J., Maji A., Dhakan D.B., Saxena R., Mohan B., Anto M.M., Agarwal N., Grace T., Sharma V.K. (2018). Gut Microbial Dysbiosis in Indian Children with Autism Spectrum Disorders. Microb. Ecol..

[25] Rosenfeld C.S. (2015). Microbiome Disturbances and Autism Spectrum Disorders. Drug Metab. Dispos..

[26] Dhamayanti M., Noviandhari A., Supriadi S., Judistiani R.T., Setiabudiawan B. (2019). Association of maternal vitamin D deficiency and infants’ neurodevelopmental status: A cohort study on vitamin D and its impact during pregnancy and childhood in Indonesia. J. Paediatr. Child Health.

[27] Dimidi E., Christodoulides S., Scott S.M., Whelan K. (2017). Mechanisms of Action of Probiotics and the Gastrointestinal Microbiota on Gut Motility and Constipation. Adv. Nutr..

[28] Zhao Y., Yu Y.-B. (2016). Intestinal microbiota and chronic constipation. Springerplus.

[29] Hsiao E.Y., McBride S.W., Hsien S., Sharon G., Hyde E.R., McCue T., Codelli J.A., Chow J., Reisman S.E., Petrosino J.F. (2013). Microbiota Modulate Behavioral and Physiological Abnormalities Associated with Neurodevelopmental Disorders. Cell.

[30] De Angelis M., Piccolo M., Vannini L., Siragusa S., De Giacomo A., Serrazzanetti D.I., Cristofori F., Guerzoni M.E., Gobbetti M., Francavilla R. (2013). Fecal Microbiota and Metabolome of Children with Autism and Pervasive Developmental Disorder Not Otherwise Specified. PLoS ONE.

[31] Tomova A., Husarova V., Lakatosova S., Bakos J., Vlkova B., Babinska K., Ostatnikova D. (2015). Gastrointestinal microbiota in children with autism in Slovakia. Physiol. Behav..

[32] Williams B.L., Hornig M., Parekh T., Lipkin W.I. (2012). Application of Novel PCR-Based Methods for Detection, Quantitation, and Phylogenetic Characterization of Sutterella Species in Intestinal Biopsy Samples from Children with Autism and Gastrointestinal Disturbances. mBio.

[33] Flint H.J., Scott K.P., Duncan S.H., Louis P., Forano E. (2012). Microbial degradation of complex carbohydrates in the gut. Gut Microbes.

[34] Henderson S.T. (2004). High carbohydrate diets and Alzheimer’s disease. Med. Hypotheses.

[35] Hellenbrand W., Boeing H., Robra B.-P., Seidler A., Vieregge P., Nischan P., Joerg J., Oertel W., Schneider E., Ulm G. (1996). Diet and Parkinson’s disease II: A possible role for the past intake of specific nutrients: Results from a self-administered food-frequency questionnaire in a case-control study. Neurology.

[36] Abbott R.D., Ross G.W., White L.R., Sanderson W.T., Burchfiel C.M., Kashon M., Sharp D.S., Masaki K.H., Curb J.D., Petrovitch H. (2003). Environmental, life-style, and physical precursors of clinical Parkinson’s disease: Recent findings from the Honolulu-Asia Aging Study. J. Neurol..

[37] Chen Y.-H., Wang H. (2020). The Association between Depression and Gastroesophageal Reflux based on Phylogenetic Analysis of miRNA Biomarkers. Current Medicinal Chemistry.

[38] Nor N.K., Ghozali A.H., Ismail J. (2019). Prevalence of Overweight and Obesity Among Children and Adolescents With Autism Spectrum Disorder and Associated Risk Factors. Front. Pediatr..

[39] Ley R.E. (2010). Obesity and the human microbiome. Curr. Opin. Gastroenterol..

[40] Gibbons H., O’Gorman A., Brennan L. (2015). Metabolomics as a tool in nutritional research. Curr. Opin. Lipidol..

[41] Ley R.E., Turnbaugh P.J., Klein S., Gordon J.I. (2006). Human gut microbes associated with obesity. Nat. Cell Biol..

[42] Xu Z., Knight R. (2015). Dietary effects on human gut microbiome diversity. Br. J. Nutr..

[43] Hosseinian F., Oomah B.D., Campos-Vega R. (2016). Dietary Fibre Functionality in Food and Nutraceuticals: From Plant to Gut.

[44] Martínez I., Lattimer J.M., Hubach K.L., A Case J., Yang J., Weber C.G., A Louk J., Rose D.J., Kyureghian G., A Peterson D. (2012). Gut microbiome composition is linked to whole grain-induced immunological improvements. ISME J..

[45] Russell W.R., Gratz S.W., Duncan S.H., Holtrop G., Ince J., Scobbie L., Duncan G., Johnstone A.M., Lobley G.E., Wallace R.J. (2011). High-protein, reduced-carbohydrate weight-loss diets promote metabolite profiles likely to be detrimental to colonic health. Am. J. Clin. Nutr..

[46] Johnson E.L., Heaver S.L., Walters W.A., Ley R.E. (2017). Microbiome and metabolic disease: Revisiting the bacterial phylum Bacteroidetes. J. Mol. Med..

[47] Khalid N., Aslam Z., Kausar F., Irshad H., Anwer P. (2017). Maternal malnutrition and its kick on child growth: An alarming trim for Pakistan. J. Food Nutr. Popul. Health.

[48] Agrawal S., Rao S.C., Bulsara M.K., Patole S.K. (2018). Prevalence of Autism Spectrum Disorder in Preterm Infants: A Meta-analysis. Pediatrics.

